# Fingolimod improves diffuse brain injury by promoting AQP4 polarization and functional recovery of the glymphatic system

**DOI:** 10.1111/cns.14669

**Published:** 2024-03-08

**Authors:** Dongyi Feng, Tao Liu, Xinjie Zhang, Tangtang Xiang, Wanqiang Su, Wei Quan, Rongcai Jiang

**Affiliations:** ^1^ Department of Neurosurgery Tianjin Medical University General Hospital Tianjin China; ^2^ Tianjin Neurological Institute, Key Laboratory of Post Neuro‐injury Neuro‐repair and Regeneration in Central Nervous System, State Key Laboratory of Experimental Hematology Ministry of Education Tianjin China

**Keywords:** AQP4, cytokine array, diffuse brain injury, fingolimod, glymphatic system, immunoinflammatory response

## Abstract

**Background:**

Diffuse brain injury (DBI) models are characterized by intense global brain inflammation and edema, which characterize the most severe form of TBI. In a previous experiment, we found that fingolimod promoted recovery after controlled cortical impact injury (CCI) by modulating inflammation around brain lesions. However, it remains unclear whether fingolimod can also attenuate DBI because of its different injury mechanisms. Furthermore, whether fingolimod has additional underlying effects on repairing DBI is unknown.

**Methods:**

The impact acceleration model of DBI was established in adult Sprague–Dawley rats. Fingolimod (0.5 mg/kg) was administered 0.5, 24, and 48 h after injury for 3 consecutive days. Immunohistochemistry, immunofluorescence analysis, cytokine array, and western blotting were used to evaluate inflammatory cells, inflammatory factors, AQP4 polarization, apoptosis in brain cells, and the accumulation of APP after DBI in rats. To evaluate the function of the glymphatic system (GS), a fluorescent tracer was injected into the cistern. The neural function of rats with DBI was evaluated using various tests, including the modified neurological severity score (mNSS), horizontal ladder‐crossing test, beam walking test, and tape sensing and removal test. Brain water content was also measured.

**Results:**

Fingolimod administration for 3 consecutive days could reduce the levels of inflammatory cytokines, neutrophil recruitment, microglia, and astrocyte activation in the brain following DBI. Moreover, fingolimod reduced apoptotic protein expression, brain cell apoptosis, brain edema, and APP accumulation. Additionally, fingolimod inhibited the loss of AQP4 polarization, improved lymphatic system function, and reduced damage to nervous system function. Notably, inhibiting the GS weakened the therapeutic effect of fingolimod on the neurological function of rats with DBI and increased the accumulation of APP in the brain.

**Conclusions:**

In brief, these findings suggest that fingolimod alleviates whole‐brain inflammation and GS system damage after DBI and that inhibiting the GS could weaken the positive effect of fingolimod on nerve function in rats with DBI. Thus, inhibiting inflammation and regulating the GS may be critical for the therapeutic effect of fingolimod on DBI.

## Graphical abstract

Diffuse brain injury‐induced inflammatory storm and glymphatic system injury in the rat nervous center can result in nerve function impairment. Fingolimod may alleviate nerve damage by modulating inflammation and reducing damage to the glymphatic system.

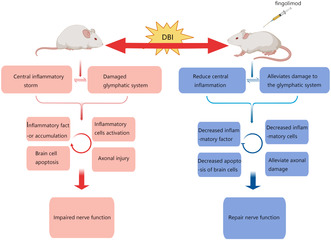

## 1 INTRODUCTION

Traumatic brain injuries (TBIs) are now considered major causes of fatalities and impairments by the World Health Organization (WHO).^1^ Since road traffic accidents and falls are the primary causes of TBIs, they have severe consequences for global health systems, national economies, families, and communities. Many fatalities related to TBIs are caused by diffuse brain injury (DBI).^1^, ^2^ This study used Marmarou's model to replicate DBI in patients since the mechanism and injury pathology differ among various brain injury models.^3^, ^4^ A statistically significant difference was observed in the extent of brain tissue damage caused by controlled cortical impact (CCI) compared with that observed in Marmarou's model for inducing DBI. CCI causes focal damage, whereas DBI causes diffuse damage.^4^ Moreover, DBI affects not only the cerebral cortex but also the cerebellum and brainstem, leading to systemic brain inflammation and edema.^2^, ^3^, ^4^ DBI causes changes in vital signs and loss of consciousness, and the mortality rate is much higher than that associated with CCI.^4^, ^5^ CCI results in wound bleeding, focal tissue deformation, and increased autophagosomes, as observed by pathological analyses. Conversely, DBI is pathologically characterized by diffuse degeneration and white matter bleeding.^6^, ^7^

Injury initiates an inflammatory response cascade involving the accumulation of necrotic cells, free radicals, toxic proteins, inflammatory cytokines, and other mediators. Furthermore, inflammation induces an influx of immune cells, which exacerbate secondary injury.^8^, ^9^, ^10^ Within a short time after injury, neutrophils are rapidly mobilized to the affected region of the brain. These cells amplify the immune response, releasing copious amounts of chemokines, proteases, and free radicals, which in turn aggravate secondary brain damage.^11^ Additionally, resident microglia are activated, and high levels of pro‐inflammatory cytokines and cytotoxic mediators are produced by M1‐subtype microglia, resulting in further neuronal damage and cell death.^12^ Previous studies have highlighted the crucial role of inflammatory reactions in generalized brain edema, axonal damage, and neuronal damage.^13^, ^14^ Furthermore, the central nervous system (CNS) is highly susceptible to the accumulation of inflammatory substances. A persistent and poorly adaptive immune response resulting from delayed clearance of inflammatory substances can lead to various CNS diseases.^15^, ^16^ Peripheral tissues rely on dense lymphatic vessels to clear metabolic waste via the lymphatic system, and the CNS has a similar mechanism called the glymphatic system (GS).^17^, ^18^, ^19^ The GS facilitates fluid exchange between cerebrospinal fluid (CSF) and interstitial fluid (ISF), aiding in the clearance of metabolic waste from the brain into the CSF and ultimately helping to discharge substances from the brain.^20^, ^21^ The GS is governed by the expression of aquaporin‐4 (AQP4) in the periarterial and perivascular space, and the loss of perivascular AQP4 polarization attenuates the function of the GS. This leads to the accumulation of toxic metabolites in a variety of CNS diseases, including traumatic brain injury, Alzheimer's disease, and stroke, ultimately resulting in neurobehavioral defects that exacerbate neurological diseases.^22^, ^23^, ^24^, ^25^

Fingolimod, which is an immunomodulatory drug, has been shown to reduce inflammation by activating the sphingosine 1‐phosphate (S1P) receptor, which decreases lymphocyte output.^26^ Research has shown that fingolimod treatment for 3 days can protect against nerve injuries by reducing the infiltration of inflammatory cells and the secretion of inflammatory factors.^27^, ^28^, ^29^ Our team revealed that fingolimod administration for 3 days could reduce the levels of several inflammatory cytokines, regulate immune cells, and improve neurological function in mice with CCI.^30^ Both DBI and CCI can lead to secondary craniocerebral injury mediated by multiple factors.^3^, ^30^ Fingolimod is involved in the treatment of CCI, but its effect may not be solely mediated by immune regulation. Thus, we hypothesized that fingolimod (0.5 mg/kg for 3 days)^26^ could alleviate nerve damage caused by DBI by regulating the inflammatory response and GS function. We evaluated the effect of fingolimod on axonal injury after DBI, inflammatory cell infiltration, the secretion of 13 cytokines, and GS function.

## 2 MATERIALS AND METHODS

### 2.1 Animals

We used Sprague–Dawley rats weighing 250–300 g, which were obtained from the Experimental Animal Laboratories of the Academy of Military Medical Sciences in Beijing, China. The rats were housed in animal facilities at Tianjin Medical University General Hospital and provided food and water ad libitum. The rats were separated into four groups: sham (saline injection, no DBI), DBI (saline injection, DBI), DBI + fingolimod (fingolimod injection, DBI), and DBI TGN‐020 (MCE, HY‐W008574, China) + fingolimod (fingolimod injection, TGN‐020 injection, DBI). The Chinese Small Animal Protection Association approved the experimental procedures, and guidelines were strictly followed. All procedures complied with the ARRIVE guidelines.

### 2.2 Diffuse brain injury model

Diffuse brain injury was induced in accordance with Marmarou's protocol.^31^ Briefly, the animals were anesthetized and stereotactically positioned, after which the skull was exposed. After the “helmet” was fixed to the parietal bone, a 450 g weight was dropped onto the helmet, causing DBI. After the trauma, mechanical ventilation with lung‐protective settings (tidal volume, 6 mL/kg body weight) was used to provide temporary respiratory assistance, and the scalp incision was closed using interrupted 6‐0 silk sutures when spontaneous ventilation was achieved. The animals were kept in heated cages for 45 min after the injury to maintain a normal temperature.

### 2.3 Drug administration

The rats in the DBI + fingolimod group (*n* = 42) were intraperitoneally injected with 0.5 mg/kg body weight fingolimod (dissolved in 0.9% sodium chloride; Cayman Chemical, 10006292) daily for 3 consecutive days beginning 30 min after the DBI procedure according to prior research and our initial results.^32^ The rats in the sham (*n* = 42) and DBI groups (*n* = 42) were injected with equivalent amounts of 0.9% sodium chloride.

TGN‐020 is known to inhibit the expression of AQP4 M23, which leads to the disruption of astrocytic AQP4 polarization.^33^ In the DBI + fingolimod + TGN‐020 group (*n* = 18), before fingolimod was administered, TGN‐020 was used to pharmacologically inhibit AQP4 polarity as previously described.^34^

### 2.4 Modified neurological severity score

The modified neurological severity score (mNSS) was used to assess functional impairment as previously described.^35^ After the motor, sensory, and reflex tests were completed, the mNSS was determined on a scale ranging from 0 to 18, with zero representing normal function and 18 indicating the maximum deficit. A higher score corresponded to more severe sensorimotor function impairment. Two observers who were blinded to the experimental conditions conducted the tests on the mice on the 1st, 3rd, 7th, and 14th days after DBI.

### 2.5 Horizontal ladder‐crossing test

The present study investigated the locomotor function of the rats on a horizontal ladder 0.9 m in length and 15.5 cm in width, which could be randomly modified for clearance.^36^ The mean error rate was computed by tallying the total number of steps taken by the rats across the ladder and recording the number of slips they made with the left and right rear paws. Subsequently, the numbers of slips and steps were used to calculate the average error rate.

### 2.6 Beam walking test

During this experiment, the rats were precisely situated at the center of a 2 m × 2 cm beam that was hanging between two elevated platforms that were approximately 60 cm above the surface. The beam walking test was used to measure walking and balance performance based on the following criteria: (1) walking normally along the beam for a minimum of 1 m, (2) crawling across the beam for at least 1 m while keeping the abdomen in contact with the beam, and (3) remaining on the beam without moving but experiencing difficulty maintaining balance.^37^

### 2.7 Tape sensing and removal test

Tape sensing and removal tests were used to evaluate the sensation of touch in the left hind paws of the rats. The rats were restrained by holding both hind limbs, and the tape was applied to the left hind paw (Kip Hochkrepp, Bocholt, Germany). The time required for the rats to locate and remove the tape was measured.^36^

### 2.8 Cerebral cortical perfusion

After the rats were fully anesthetized, cortical blood perfusion was examined 3 days after DBI using a laser speckle imager (PeriCam PSI system, Peried AB, Sweden). The height was 10 cm, the laser irradiation area was 2 × 2 cm, and the PSI system was set at 1388 × 1038 pixels. The region space contrast was calculated according to the 3 × 3 matrix. Cortical blood perfusion was measured continuously for 30 s. After the results were averaged, the perfusion data were evaluated using PIMsoft (version 1.4).^38^

### 2.9 Brain water content

Brain water content was quantified by determining the ratio of the wet brain weight (WW) to the dry brain weight (DW) after 3 days of DBI. To obtain the WW, brain samples were collected following deep anesthesia and weighed using an electronic analytical balance. The DW was measured by the same method after the samples were dried at 100°C for 24 h. Brain water content was calculated using the formula (WW − DW)/WW.^16^

### 2.10 Immunohistochemical staining

The rats were fully anesthetized after 3 days of DBI and injected with PBS mixed with 4% paraformaldehyde (PFA) at 4°C. The brains were subsequently removed, fixed, and embedded in paraffin. Adequately spaced coronal sections with a distance of 8 μm were prepared.

To determine the extent of nerve damage, amyloid precursor protein (APP) immunostaining was performed on the brain sections. Neutrophil infiltration was assessed by myeloperoxidase (MPO) immunostaining. The paraffin‐embedded sections were incubated at 60°C in a constant temperature air box for 2.5 h. Subsequently, the paraffin was removed from these sections step by step. The sections were treated with 0.3% H_2_O_2_ for 30 min at room temperature (RT) to inactivate endogenous peroxidases. Antigen retrieval was performed by boiling the sections for 10 min in 10 mM citrate buffer (pH 6.0). Nonspecific binding was blocked for 30 min with 1% BSA in PBS. The tissue sections were stained with β‐APP or anti‐MPO antibodies. Primary antibodies against rabbit anti‐APP (1:500, Abcam, ab32136) and anti‐myeloperoxidase (1:500, Abcam, ab9535) were added and incubated overnight at 4°C, followed by the application of a secondary biotinylated goat anti‐rabbit antibody (1:100, Santa Cruz Biotechnology) for 1 h at room temperature (RT). The sections were subsequently incubated for 1 h at RT with an avidin peroxidase conjugate solution (1:100, Santa Cruz Biotechnology). Negative controls were prepared using the same procedure. The extent of APP and MPO accumulation was measured using ImageJ software (version 1.48v, NIH).

### 2.11 Immunofluorescence staining

After 3 days of DBI, the rat brains were perfused with cold PBS and quickly embedded in OCT medium (Sakura, USA), after which 8‐μm‐thick sections were prepared. The sections were subsequently fixed in cold acetone at 4°C for 5 min, blocked with 3% BSA at room temperature for 30 min, and then incubated with the following primary antibodies at 4°C overnight: Iba‐1 (1:500, Abcam, catalog number: ab5076), AQP4 (1:500, Cell Signaling Technology, catalog number: 59678T), CD31 (1:500, R&D Systems, catalog number: AF3628), and GFAP (1:500, Abcam, catalog number: ab48004). Then, the sections were incubated with Tritc‐conjugated anti‐rabbit IgG (1:100; Zsgb‐bio) at room temperature for 1 h in the dark. DAPI was used as a counterstain for 5 min. The negative controls were treated with PBS. Finally, the slices were visualized under a fluorescence microscope at ×200 magnification, and the positive cells in matched sections were counted using ImageJ software (version 1.48v, NIH).

Brain cell apoptosis was evaluated using a fluorometric terminal deoxynucleotidyl transferase deoxyuridine triphosphate (dUTP) nick end labeling (TUNEL) system (Promega, Madison, WI). Negative controls were prepared with the same procedures but without the primary antibody. The positive areas of polarized light microscopy with second harmonic generation (PSR) were quantified using ImageJ software.

### 2.12 Evaluating AQP4 polarization

The relative position of AQP4 was measured 3 days after DBI to evaluate AQP4 polarization. As previously mentioned,^39^ the immunofluorescence intensity in the perivascular area was measured to establish a threshold. This threshold was subsequently used to determine the percentage of the AQP4 immunofluorescence region that was equal to or greater than the perivascular AQP4 immunofluorescence region, which was referred to as the AQP4% area. AQP4 polarization was calculated as 100 minus the AQP4% area.

### 2.13 Cytokine array

After 3 days of DBI, we fully perfused the brain tissues with ice‐cold PBS and homogenized the tissue with PBS containing a protease inhibitor. We then centrifuged the samples at 4°C and 10,000 × *g* for 5 min. We used the Solaribo (BCA) method to determine the total protein concentration in the samples. Inflammatory cytokines, including CXCL‐1, CXCL‐7, CXCL‐9, CXCL‐5, CX3CL1, IL‐1a, IL‐1b, IL‐1ra, IL‐3, IL‐6, IL‐10, TNF‐α, and VEGF, were assessed using Rat Cytokine Array Panel A (ARY008, R&D Systems, Minneapolis, MN, USA) according to the protocol. The protein (200 μg) was mixed with the biotinylated detection antibody and incubated with the membrane at 4°C overnight. The next day, the membrane was washed thoroughly with 1× washing buffer solution and incubated with streptavidin HRP on a shaker at room temperature for 30 min. After further washes, the membrane is exposed to the ECL system (Millipore, Billerica, MA). We repeated these experiments three times. Finally, we measured the expression of inflammatory cytokines and chemokines using Quantity One software (version 4.6.2; Bio‐Rad) and averaged the pixel density. We corrected the spot density of a single background to reduce variation between the arrays.

### 2.14 Western blotting

After 3 days of DBI, the rats were fully infused with ice‐cold PBS, and their brains were harvested. The brain tissue was homogenized in radioimmunoprecipitation assay (RIPA) lysis buffer, and the resulting supernatant was mixed with loading buffer until a concentration of 5 μg/mL was achieved. Equal amounts of protein samples (30 μg) were loaded onto 7.5%–12% sodium dodecyl sulfate–polyacrylamide gel electrophoresis (SDS–PAGE) gels, separated by electrophoresis and subsequently transferred onto nitrocellulose membranes (0.22 μm). The membranes were incubated with blocking buffer (5% nonfat milk) for 1 h at room temperature and then with primary antibodies overnight at 4°C. The primary antibodies used included anti‐β‐actin (ab8227, Abcam), anti‐Bcl‐2 (1:1000, WLO1556, Wanleibo), anti‐caspase‐3 (ab13847, Abcam), anti‐Bax (ab32503, Abcam), anti‐AQP4 (59678T, Cell Signaling Technology, Danvers, MA), anti‐APP (ab32136, Abcam), and anti‐GFAP (ab7260, Abcam). The following day, the corresponding secondary antibodies were added and incubated with the membranes at room temperature for 1 h. Finally, the immunoblots were visualized using an ECL Plus chemiluminescence reagent kit and quantified using Fiji (ImageJ) software.

### 2.15 Intracisternal tracer infusions

A fluorescent tracer was used to visualize the influx of cerebrospinal fluid through the perivascular space into the cerebral parenchyma and the movement of cerebrospinal fluid and lymph out of the brain as described. After 3 days of DBI, the rat was anesthetized, and the head was secured on a stereoscopic frame. An incision was made in the midline of the skin to expose the cisterna magna. To deliver the tracer, 40 μL of rhodamine B isothiocyanate (RITC) conjugated with dextran (molecular weight 70 kDa; Sigma, 2.5% in artificial CSF) was injected into the cisterna magna with a Hamilton syringe. The tracer was allowed to circulate for 30 min, after which the animals were euthanized and infused with phosphate‐buffered saline (PBS; pH 7.4; Solarbio). Next, the brain tissue was removed, fixed in 4% paraformaldehyde in PBS overnight, dehydrated with 30% sugar for 2 days, and frozen with OCT (Thermo Fisher Scientific). The brain was sliced into 100 μm coronal sections using a Leica Microsystems instrument. A total of three sections representative of the craniocaudal axis were acquired and then stained with DAPI for 30 min before being placed on coverslips for fluorescence imaging.

### 2.16 Statistical analysis

The data were analyzed using SPASS 21 software and one‐way analysis of variance (ANOVA) with a least significant difference (LSD) post hoc test or an unpaired *t* test as applicable. The mean values are expressed as the mean ± standard error of the mean (SEM). A *p*‐value of less than 0.05 indicated statistical significance.

## 3 RESULTS

### 3.1 Fingolimod‐ameliorated neurological deficits, cerebral perfusion, and brain edema after DBI

**FIGURE 1:**
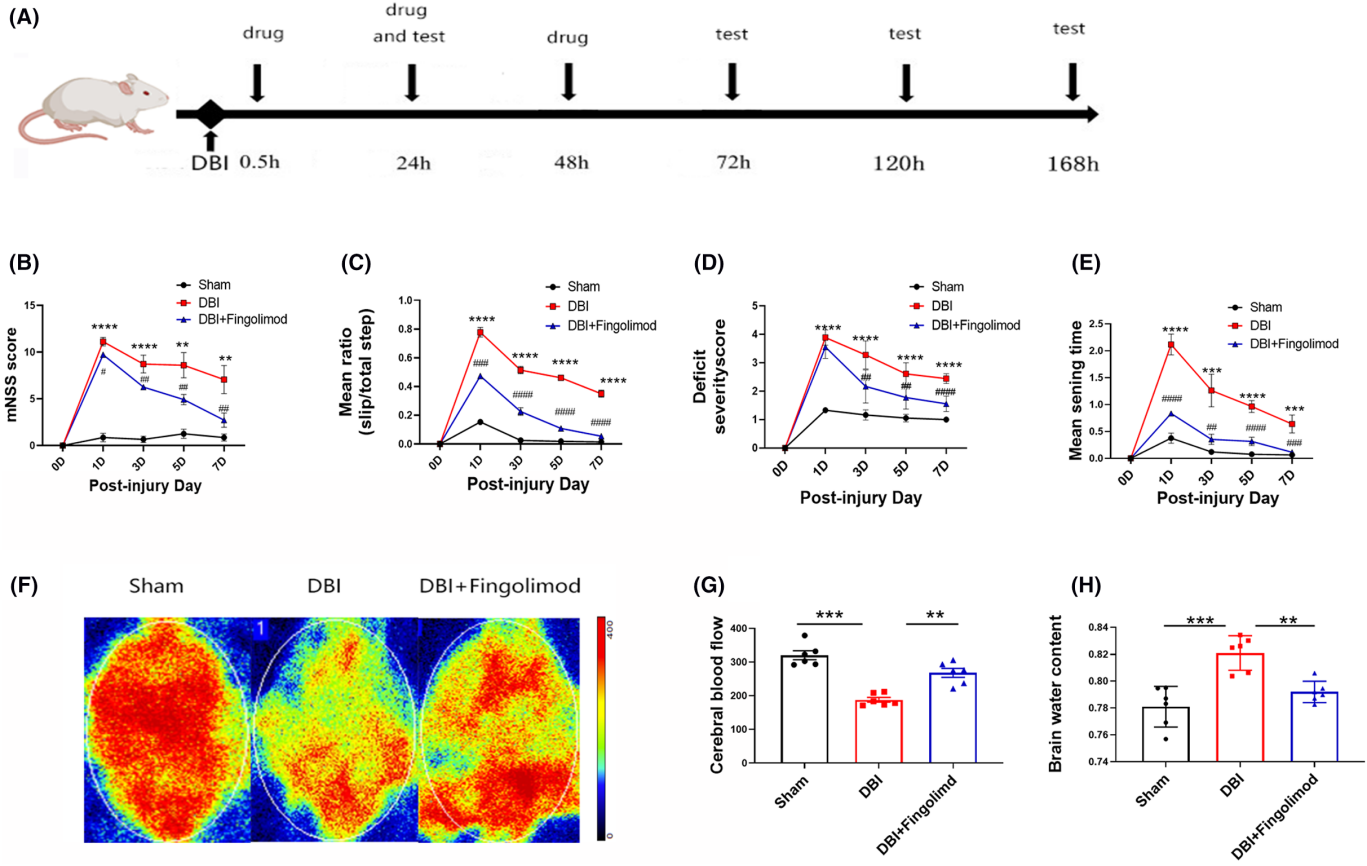
The effects of fingolimod on neurological outcomes, cerebral cortical blood perfusion, and brain edema after DBI. (A) Experimental timeline, (B) neurological functions were evaluated by determining the mNSS, (C) ladder‐crossing test, (D) beam walking, (E) mean sensing time for the tape removal test, (F, G) representative laser speckle contrast images and quantitative analyses of cerebral cortical blood perfusion in rats 3 days after DBI. (H) The brain water content of rats 3 days after DBI (*n* = 6 per group; three independent repeats; total *n* = 18 rats). The data are shown as the mean ± SEM. **p < 0.01, ***p < 0.005, ****p < 0.001, ^#^ *p* < 0.05, ^##^ *p* < 0.01, ^###^ *p* < 0.005, ^####^ *p* < 0.001. One‐way ANOVA, Tukey's post hoc test. DBI, diffuse brain injury; mNSS, modified neurological severity score.

As shown in Figure 1A, we examined the potential benefits of fingolimod on neurological deficits, locomotor function, and sensory recovery in rats subjected to DBI. We used the modified neurological severity score (mNSS) to assess functional impairment.^35^ Additionally, we conducted the horizontal ladder‐crossing and beam walk tests to gauge locomotor function. During the former test, the rats traversed a 0.9 m ladder with rungs randomly spaced 3.5–5.0 cm apart, and the number of slips/footfalls out of the total number of steps taken to cross was recorded.^36^ The latter test enabled us to discern how well the injured rats could maintain balance and walk on a narrow beam.^37^ Finally, we used the tape sensing and removal test to evaluate sensory recovery. During this test, a small piece of adhesive tape was placed on the hind paw, and we recorded the time taken for the tape to be detected and removed.^36^

All rats in the DBI group exhibited motor neurological severity scores (mNSS) on the 1st day than those in the sham group (Figure 1B). However, on subsequent days, neurological deficits started to recover in the groups. Fingolimod‐treated rats exhibited a lower mNSS on the following days than rats in the DBI group (Figure 1B). One day postinjury, there was a significant deficit in ladder‐crossing performance in all groups, which persisted for 1 week in the DBI group (Figure 1C). By the 7th day after injury, fingolimod treatment resulted in significant improvements in ladder‐crossing performance, and the rats were indistinguishable from those in the sham group (Figure 1C). Similarly, all DBI‐treated rats exhibited significant impairment in the beam walking test; rats in the DBI group were unable to balance or remain on the beam 1 day after injury, while those in the fingolimod group could remain on the beam (Figure 1D). On subsequent days, walking and balancing deficits started to recover in all the groups, and the DBI group had a significantly greater deficit than the fingolimod group (Figure 1D). By the 7th day postinjury, there was no significant difference in the results of the beam walking test between the sham and fingolimod groups, while the DBI group still exhibited significant deficits in walking and balancing (Figure 1D). Postinjury, the mean time for rats with DBI to sense and remove the adhesive tape was longer than that in the sham group. However, the increase was significantly less pronounced in the fingolimod group than in the DBI group (Figure 1E). In the sham animals, the mean sensing and removal times ranged between 12 and 29 s throughout the assessment period, and the fingolimod group showed significant improvements during this period. Conversely, the DBI group exhibited significant deficits at 7 days postinjury, and animals took an average of 38 s to sense and remove the tape (Figure 1E).

The results showed that DBI significantly decreased cerebral cortical perfusion and induced a concomitant increase in brain edema on the 3rd day postinjury (refer to Figure 1F–H). In contrast, fingolimod treatment significantly increased cerebral cortical perfusion and decreased the brain water percentage compared to those in the DBI group on the 3rd day after injury (Figure 1F–H).

### 3.2 Fingolimod decreased the number of activated microglia and neutrophils, GFAP, inflammatory cytokines, and axonal damage after DBI

**FIGURE 2:**
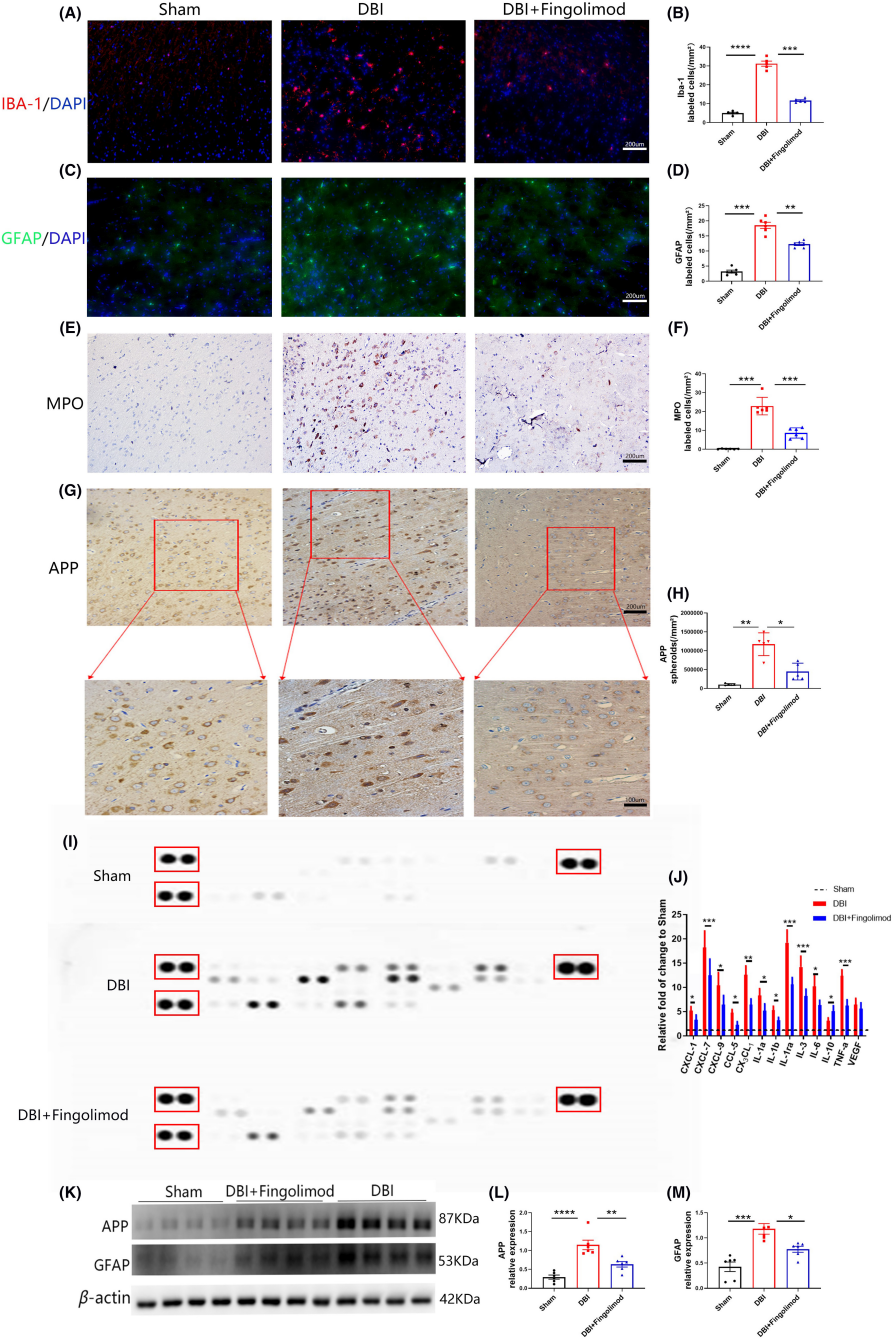
Effects of fingolimod on inflammatory cells, inflammatory factors, and axonal damage after DBI. (A, B) Representative images and statistical analysis of immunofluorescence staining of microglia (red) in brain sections obtained 3 days after DBI. Scale bar: 200 μm. (C, D) Representative images and statistical analysis of immunofluorescence staining of astrocytes (green) in brain sections obtained 3 days after DBI. Scale bar: 200 μm. (E, F) Representative images and statistical analysis of immunofluorescence staining of neutrophils in brain sections obtained 3 days after DBI. Scale bar: 200 μm. (G, H) Representative images and statistical analysis of immunofluorescence staining of APP 3 days after DBI. Scale bar: 200 μm. (I, J) Representative images of the inflammatory cytokine array and statistical analysis of the pixel density of each marker in the array. (K–M) Representative western blots and densitometric analysis of the expression of APP and GFAP 3 days after DBI (*n* = 5–6 per group; three independent repeats; total *n* = 15–18 rats). The data are shown as the mean ± SEM. *p < 0.05, **p < 0.01, ***p < 0.005, ****p < 0.001. One‐way ANOVA, Tukey's post hoc test. APP, amyloid precursor protein; DBI, diffuse brain injury; GFAP, glial fibrillary acidic protein; IBA‐1, ionized calcium‐binding adapter molecule‐1; MPO, myeloperoxidase.

Diffuse brain injury significantly increased the number of activated microglia, astrocytes and neutrophils, and fingolimod substantially reduced the total activation of these cells (Figure 2A–F). Immunohistochemical staining was performed using an APP antibody, which is widely used to assess axonal damage.^39^ Significant accumulation of APP was observed 3 days after DBI, and the accumulation of APP was considerably decreased in the fingolimod treatment group compared with the DBI group (Figure 2G,H). On the 3rd day after DBI, fingolimod treatment reduced the expression of 13 cytokines and chemokines, including CXCL‐1, CXCL‐7, CXCL‐9, CCL‐5, CX3CL1, IL‐1a, IL‐1b, IL‐1ra, IL‐3, IL‐6, IL‐10, TNF‐α, and VEGF, compared with those in the DBI group (Figure 2I,J). Specifically, IL‐10 concentrations in the fingolimod treatment group were higher than those in the DBI group, but there was no significant difference in VEGF levels between the groups (Figure 2I,J). To further validate the activation of astrocytes and APP accumulation, western blotting was performed. The findings were consistent with the previous results (Figure 2K–M).

### 3.3 Fingolimod significantly reduced apoptosis after DBI

**FIGURE 3:**
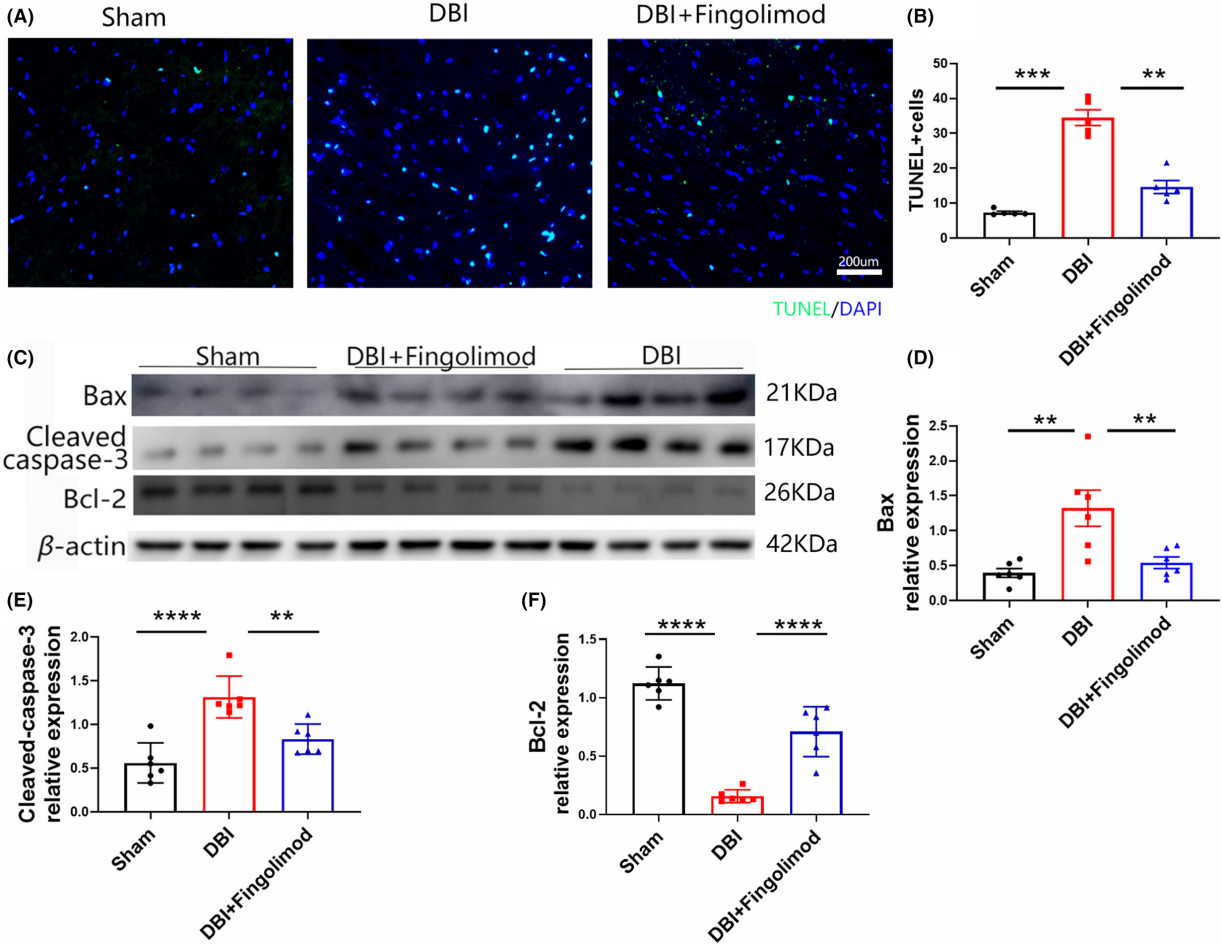
Effects of fingolimod on apoptosis in brain tissue after DBI. (A, B) Apoptosis and statistical analysis of representative TUNEL‐stained cells 3 days after DBI. (C) Representative western blots showing bax, bcl‐2, and cleaved caspase‐3 expression 3 days after DBI. (D–F) Western blot and densitometric analysis of bax, bcl‐2, and cleaved caspase‐3 in the brains of rats 3 days after DBI (*n* = 5–6 per group; three independent repeats; total *n* = 15–18 rats). The data are shown as the mean ± SEM. **p < 0.01, ***p < 0.005, ****p < 0.001. One‐way ANOVA, Tukey's post hoc test. Bax, Bcl‐2‐associated X; Bcl‐2, B‐cell lymphoma‐2; DBI, diffuse brain injury; TUNEL, terminal deoxynucleotidyl transferase‐mediated dUTP nick end labeling.

To investigate the effects of fingolimod on cerebral nerve cell apoptosis, we performed TUNEL immunostaining and western blot analysis of apoptosis‐related proteins 3 days after DBI. As shown in Figure 3A,B, rats subjected to DBI exhibited significant increases in cerebral nerve cell apoptosis and apoptosis‐related protein expression in brain cells compared with rats in the sham group. However, treatment with fingolimod significantly reduced the amount of cerebral nerve cell apoptosis. Furthermore, western blot analysis revealed reduced expression of the apoptosis‐related proteins bax, bcl‐2, and cleaved caspase‐3 (Figure 3C–F) in the fingolimod‐treated group compared with the DBI group.

### 3.4 Fingolimod‐attenuated glymphatic pathway dysfunction, increased perivascular AQP4 polarization, and reduced AQP4 expression after DBI

**FIGURE 4:**
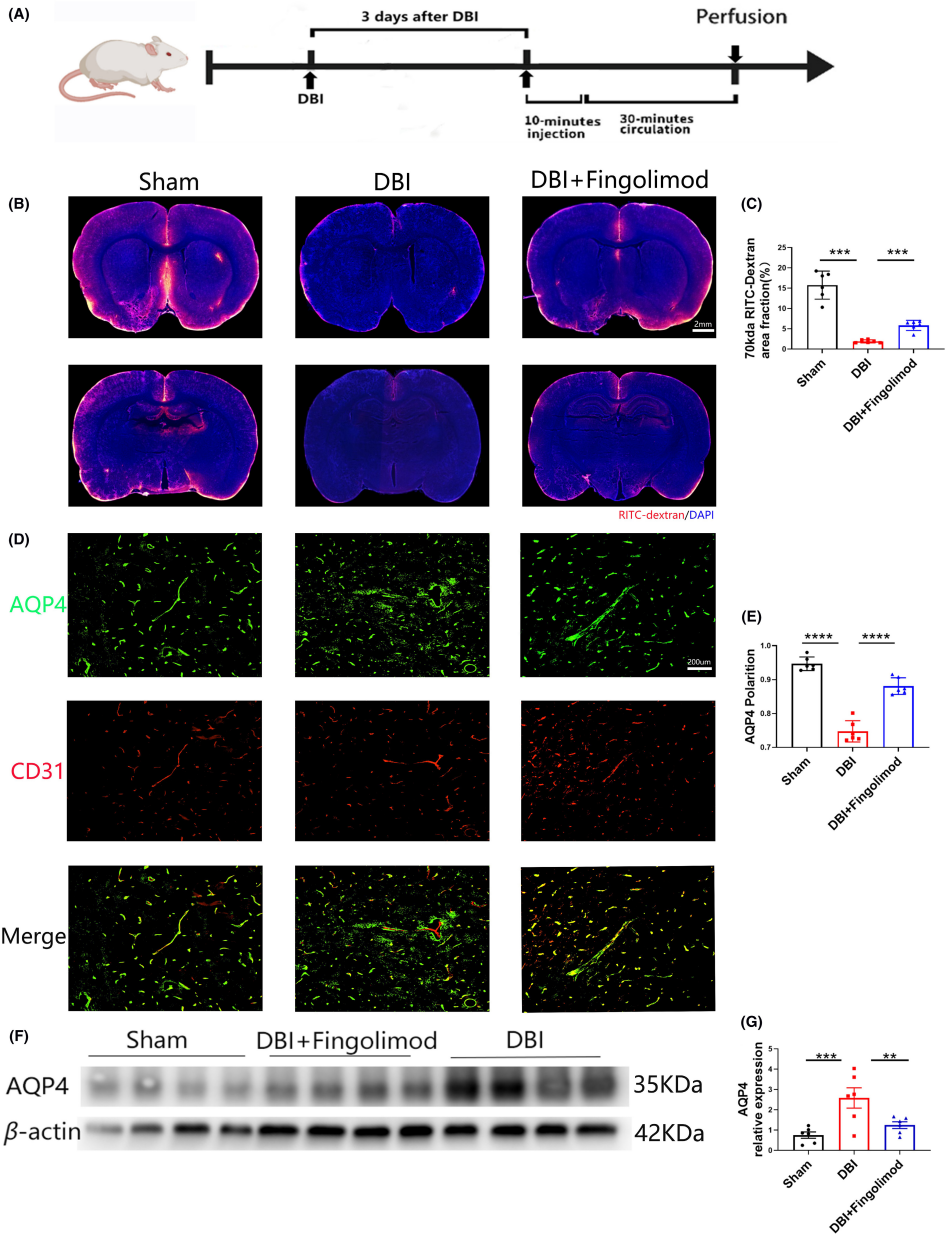
Effects of fingolimod on glymphatic system in the brain after DBI. (A) Experimental duration of fluorescent dye injection in the cisterna occipitalis. (B) Representative brain sections stained with DAPI (blue) and showing RITC‐dextran (red) influx into the brain parenchyma of rats after DBI. Scale bar: 2 mm. (C) Quantification of the percentage of the RITC‐dextran‐covered area in brain sections. (D) Representative images of immunofluorescence staining of AQP4 (green) and CD31 (red) in the brain 3 days after DBI. Scale bar: 200 μm. (E) Representative western blots showing AQP4. (F) Quantification of AQP4 polarization 3 days after DBI. (G) Densitometric analysis of AQP4 (*n* = 6 per group; three independent repeats; total *n* = 18 rats). The data are shown as the mean ± SEM. **p < 0.01, ***p < 0.005, ****p < 0.001. One‐way ANOVA, Tukey's post hoc test. AQP4, aquaporin 4; CD31, platelet endothelial cell adhesion molecule‐1; DBI, diffuse brain injury; RITC‐dextran, rhodamine B isothiocyanate‐dextran.

To evaluate whether DBI affects CSF penetration into the brain parenchyma, we infused a tracer molecule called 70 kDa RITC‐dextran into the subarachnoid CSF of the cisterna magna I 3 days after DBI and in a cohort of sham rats, as shown in Figure 4A. Thirty minutes after the CSF tracer was infused, the animals were perfusion‐fixed, their brains were sliced, and tracer penetration was evaluated using whole‐slice fluorescence imaging and in vitro fluorescence microscopy. CSF penetration into CNS structures was measured by assessing the percentage of the area occupied by RITC‐dextran in three representative slices from the groups. We defined CSF penetration by averaging the fluorescence region coverage in three representative brain slices from each rat. CSF tracer penetration into the brain was significantly lower in rats with DBI than in sham rats, suggesting that DBI can affect glymphatic circulation, as shown in Figure 4B,C. Additionally, there was a noticeable decrease in influx in the DBI group compared to the fingolimod group, indicating that fingolimod mitigated damage to glymphatic circulation, as shown in Figure 4D,E. Western blot analysis revealed reduced AQP4 expression (Figure 4F,G) in the fingolimod‐treated group compared with the DBI group.

### 3.5 TGN‐020 attenuates fingolimod‐mediated restoration of GS function in rats with DBI by inhibiting the repolarization of AQP4

**FIGURE 5:**
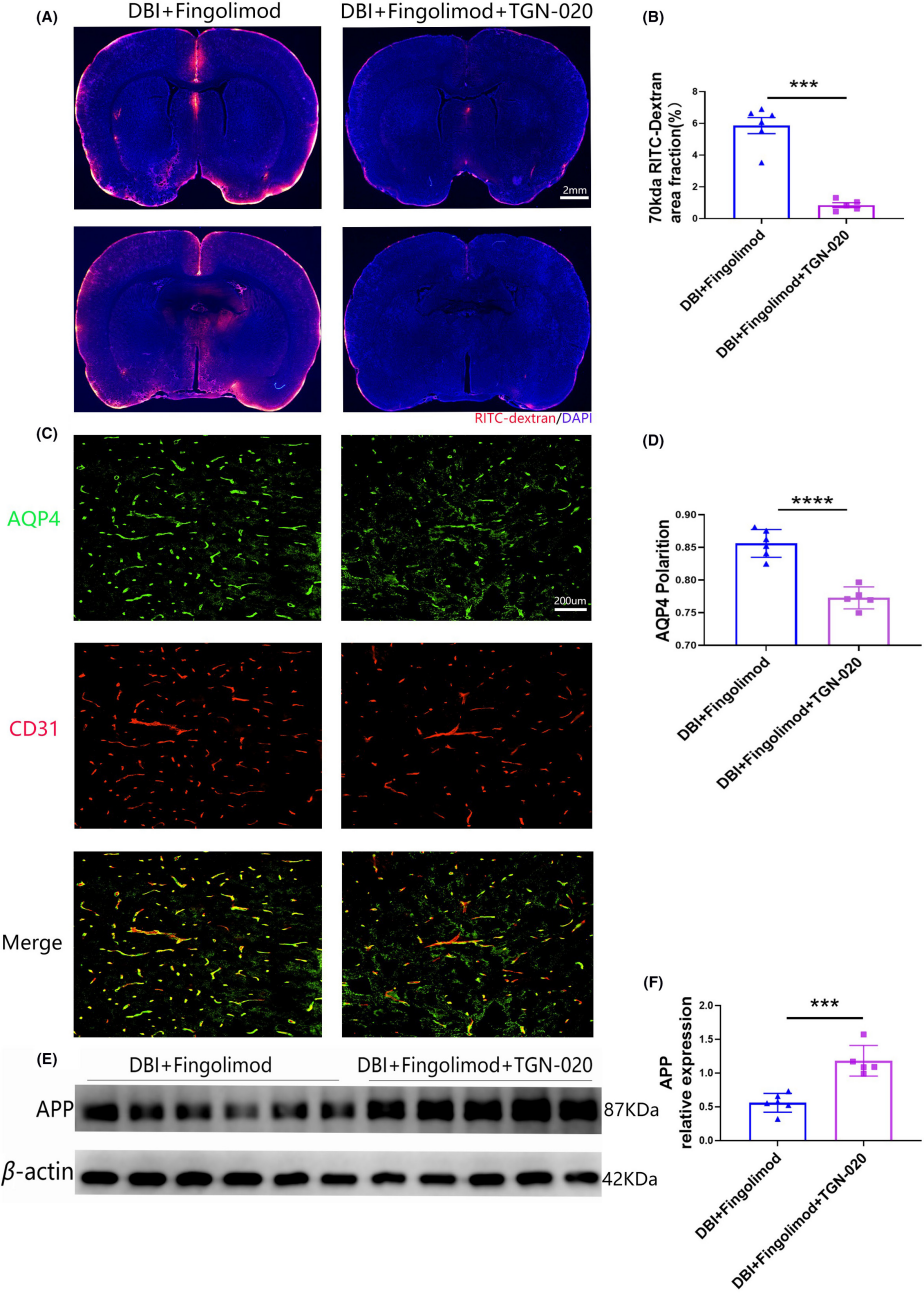
TGN‐020 blocks the therapeutic effects of fingolimod on rats with DBI by damaging glymphatic function. (A) Representative brain sections stained with DAPI (blue) and showing RITC‐dextran (red) influx into the brain parenchyma of rats after DBI. Scale bar: 2 mm. (B) Quantification of the percentage of the RITC‐dextran‐covered area in brain sections after DBI. (C) Representative images of immunofluorescence staining of AQP4 (green) and CD31 (red) in the brain 3 days after DBI. Scale bar: 200 μm. (D) Quantification of AQP4 polarization 3 days after DBI. (E) Representative western blots showing APP in the brain 3 days after DBI. (F) Densitometric analysis of APP (*n* = 5–6 per group; three independent repeats; total *n* = 15–18 rats). The data are shown as the mean ± SEM. ***p < 0.005, ****p < 0.001. One‐way ANOVA, Tukey's post hoc test. APP, amyloid precursor protein; AQP4, aquaporin 4; CD31, platelet endothelial cell adhesion molecule‐1; DBI, diffuse brain injury; RITC‐dextran, rhodamine B isothiocyanate‐dextran.

According to previous publications, TGN‐20 can disrupt astrocytic AQP4 polarization, making it a suitable inhibitor of AQP4 polarization for use in this study.^22^, ^40^ To investigate the protective effect of fingolimod following DBI, we used TGN‐020 to suppress glymphatic transport. Our results revealed a significant reduction in the penetration of the CSF tracer into the brains of rats subjected to DBI in the presence of fingolimod and TGN‐020, particularly compared with that in rats treated with fingolimod alone (Figure 5A,B). Rats with DBI that were treated with fingolimod and TGN‐020 exhibited a decrease in the perivascular polarization of AQP4 compared to that in rats treated with fingolimod alone (Figure 5C,D). Additionally, western blot analysis revealed an increase in the accumulation of APP in the brain (Figure 5E,F). These findings suggest that TGN‐020 treatment can counteract the recovery of glymphatic function induced by fingolimod, blocking its effects on rats with DBI.

### 3.6 TGN‐020 attenuated the therapeutic effect of fingolimod on nerve function in rats with DBI

**FIGURE 6:**
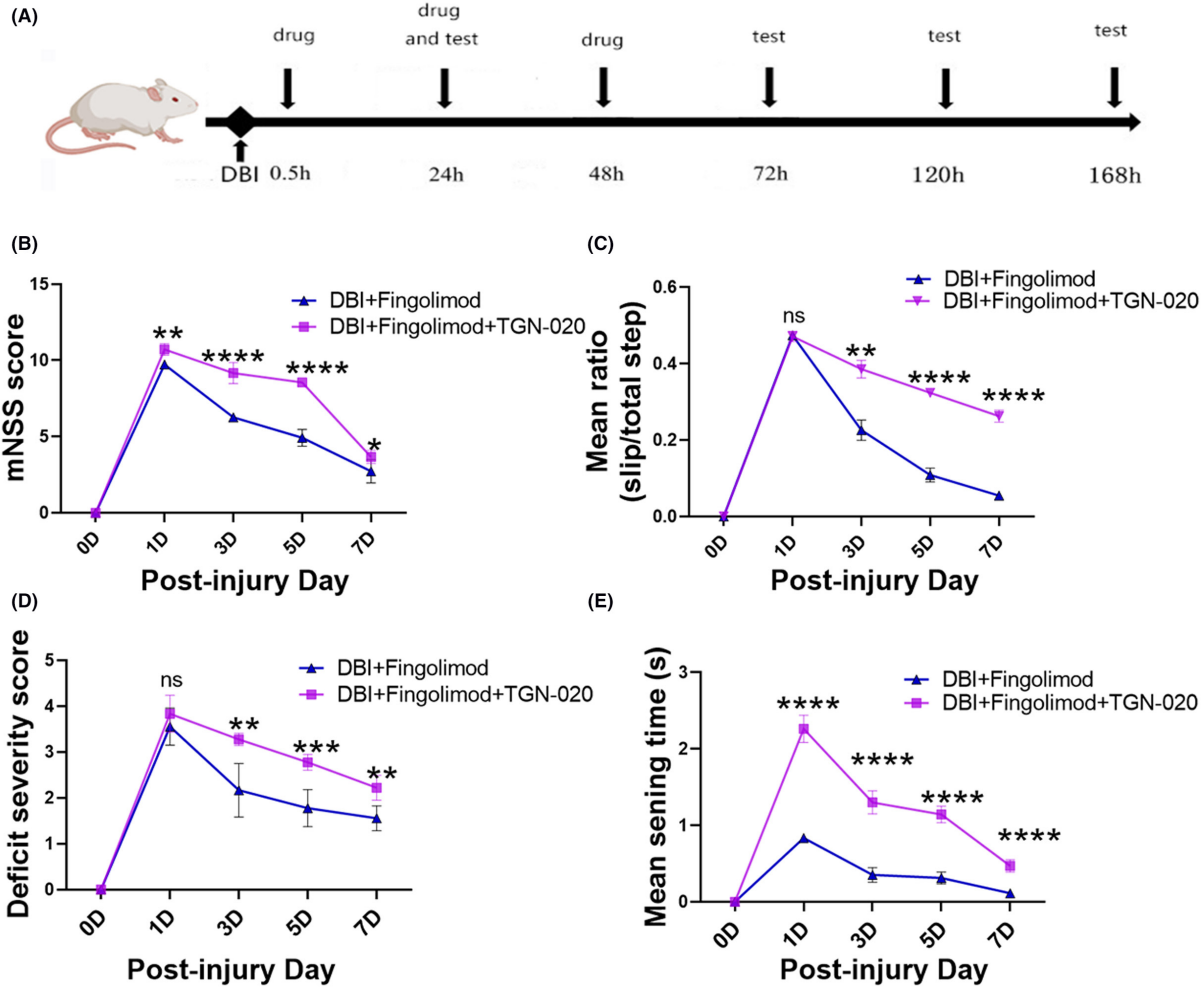
TGN‐020 blocks the therapeutic effect of fingolimod on nerve function in rats with DBI. (A) Experimental timeline, (B) neurological function was evaluated with the mNSS, (C) ladder‐crossing test, (D) beam walking test, (E) mean sensing time for the tape removal test (*n* = 5–6 per group; two independent repeats; total *n* = 10–12 rats). The data are shown as the mean ± SEM. ns: no statistical significance, *p < 0.05, **p < 0.01, ***p < 0.005, ****p < 0.001 . One‐way ANOVA, Tukey's post hoc test. DBI, diffuse brain injury; mNSS, modified neurological severity score.

As shown in Figure 6A, we investigated the effects of inhibiting AQP4 polarization on neurological deficits, motor function, and sensory recovery in rats with DBI that were treated with fingolimod. Figure 6B shows that rats with DBI had an elevated mNSS on the 1st day after injury. Subsequently, the neurological deficits started to recover. Rats treated with fingolimod+TGN‐020 had lower mNSSs on the following days than those in the fingolimod group (Figure 6B). As shown in Figure 6C, there was no significant difference in ladder leaping ability between the fingolimod group and the fingolimod + TGN‐020 group on the 1st day after DBI, but a remarkable increase in ladder leaping ability was observed in the fingolimod group and the other group on days 3, 5, and 7 after fingolimod treatment. Similarly, on the 1st day after DBI, there was no significant difference in walking or balance impairment between the fingolimod group and the fingolimod + TGN‐020 group. However, walking and balance impairment improved in the fingolimod group with time compared with that in the fingolimod + TGN‐020 group (Figure 6D). As shown in Figure 6E, the mean sensation and removal time in the fingolimod + TGN‐020 treatment group was considerably increased during all observation periods compared with that in the fingolimod + TGN‐020 group. These findings suggest that sensory recovery was inhibited in the fingolimod + TGN‐020 group.

## 4 DISCUSSION

The present study focused on fingolimod‐mediated immunomodulatory effects, improvements in GS function, and neuroprotective effects on rats after DBI, and our results showed that fingolimod decreased the production of multiple pro‐inflammatory cytokines and chemokines and the infiltration of inflammatory cells induced by DBI, thereby reducing the inflammatory response in rats. Fingolimod also reduced cerebral edema, improved cerebral blood flow, mitigated axonal damage, restored GS damage, accelerated APP metabolite clearance, and improved nerve function in rats with DBI. There is a synergy between cerebral edema and decreased cerebral blood flow after injury that can lead to increased intracranial pressure, resulting in poor clinical prognosis after DBI.^41^, ^42^ We propose that drugs that target the decrease in cerebral blood flow and cerebral edema following DBI are likely to improve neurological injury caused by DBI. Previous studies have shown that fingolimod reduces cerebral edema in neurological diseases such as cerebral hemorrhage and ischemia,^28^, ^43^ which is consistent with our observations in rats with DBI. Furthermore, our study revealed that fingolimod effectively decreased apoptosis induced by brain injury, which is consistent with the findings of previous studies on the potential of fingolimod to treat neurological conditions such as CCI and ischemia.^30^, ^44^ Our initial findings indicate that inhibiting the polarization of AQP4 using TGN‐020 impairs the therapeutic effect of fingolimod on rats with DBI. We hypothesize that promoting the polarization of AQP4 regulates the GS and that inhibiting the polarization of AQP4 reverses the therapeutic effects of fingolimod on DBI. These results suggest that fingolimod is a favorable treatment option for patients suffering from DBI, revealing novel perspectives for clinical treatments.

Cerebral infarction and multiple TBIs, including DBI, are known causes of central neuritis.^25^, ^45^ The main types of immune cells that initiate the inflammatory cascade are astrocytes and microglia.^46^ Furthermore, when these immune cells are activated by inflammatory injury, they can reduce GS clearance.^47^ Consequently, the accumulation of inflammatory cytokines in brain cells due to the obstruction of GS clearance can activate additional immune cells, further aggravating neuroinflammation and inhibiting lymphatic flow.^46^, ^47^ Likewise, lymphatic fluid flow was reduced when AQP4 was knocked out in mice.^48^ DBI causes nerve inflammation and increases the expression of AQP4,^22^ which was supported by our western blot results. However, during neuroinflammation, AQP4 is increased but is not inserted into the periarterial space of the astrocyte terminal foot but rather into cell bodies and perisynapses. This finding suggests that the increase in AQP4 expression during neuroinflammation does not support lymphatic transport.^39^ Furthermore, the increase in AQP4 induced by neuroinflammation exacerbates the loss of vascular AQP4 polarization, which indicates impaired GS function.^39^, ^49^ Additionally, during inflammation, many inflammatory cells infiltrate and accumulate in the perivascular space, which may physically block lymphatic transport.^50^, ^51^ There seems to be a mutually reinforcing relationship between an increase in neuroinflammation and GS impairment. Therefore, reducing the inflammatory response after DBI by administering fingolimod is a crucial step in disrupting this relationship and restoring GS function.

During systemic inflammation, inflammatory cytokines are known to induce excessive secretion of CSF and the activation of glial cells, leading to impaired lymphatic clearance in the brain.^47^ Fingolimod has been shown to reduce cytokine secretion and the recruitment of immune cells, consequently reducing central and peripheral inflammatory responses.^28^ Many recent studies have demonstrated that fingolimod exerts direct protective effects on nerve cells in the brain. These effects include inhibiting the activation of microglia and astrocytes, reducing the loss of dendritic spines, and restoring synaptic defects to some degree.^52^ Additionally, fingolimod can inhibit the development of reactive astrocyte hyperplasia after DBI, thereby maintaining the polarization of AQP4. It can restore lymphatic clearance and ultimately prevent posttraumatic neurodegeneration.^28^, ^52^ Therefore, fingolimod can alleviate the impairment of GS function after DBI by (1) reducing inflammatory damage caused by DBI, which activates astrocytes and microglia, thus slowing their damage to GS; (2) reducing the activation and recruitment of inflammatory cells in the nervous system after DBI, relieving the accumulation of inflammatory cells in the space around cerebral blood vessels and restoring lymphatic fluid transport; and (3) alleviating the increase in AQP4 expression in the rat brain caused by DBI, restoring the polarization of AQP4 and further restoring the clearance of cerebral lymph. In summary, fingolimod regulates inflammation in DBI and alleviates the GS damage caused by DBI.

Our study is believed to be the first to examine the effect of fingolimod on GS function in DBI. Although fingolimod has been studied as a potential treatment for DBI, experimental studies on the underlying mechanisms remain limited. Further studies are needed to further explore the feasibility of its clinical application. Recent evidence highlights the need for new treatment approaches to alleviate GS dysfunction during the progression of traumatic brain injury.^22^ This study provides a theoretical foundation for the establishment of a comprehensive treatment strategy for DBI by regulating intracranial immune inflammation and promoting GS drainage. Furthermore, this study provides new insights into the potential use of fingolimod as a treatment for patients with brain trauma.

## 5 CONCLUSION

In conclusion, our findings indicate that the administration of fingolimod for 3 days can reduce DBI‐induced brain injury by mitigating inflammatory cytokine expression in brain tissue, mitigating inflammatory cell infiltration, reducing brain cell apoptosis, preventing the loss of AQP4 polarization, and preserving GS function. These findings provide crucial insights into the clinical treatment of DBI, illuminating the role of fingolimod in alleviating this condition by regulating the inflammatory response and preserving GS function.

## 6 TRANSPARENCY, RIGOR, AND REPRODUCIBILITY SUMMARY

The study involved 144 rats divided into four cohorts, and each group consisted of 5–6 rats that were subjected to experimental DBI or sham injury. The analysis was performed by an observer who was unaware of the experimental conditions. The antibodies for TFT, WB, and FCM were procured from the same laboratory where the staining took place. Prior to that, the specificity and accuracy of the antibodies were tested through preliminary studies. The normal distribution of the original histological data was confirmed using the Kolmogorov–Smirnoff test. The data collected for this study are available upon request.

## AUTHOR CONTRIBUTIONS

DF contributed to the conception and design of the study. DF, WQ, and TX performed the experiments. DF, XZ, and TL analyzed the data, and DF wrote the first draft of the manuscript, with TX, TL, and XZ contributing to the figure design. All the authors contributed to the manuscript revision and read and approved the submitted version.

## FUNDING INFORMATION

The author(s) disclosed receipt of the following financial support for the research, authorship, and/or publication of this article. This study was supported by the National Natural Science Foundation of China (no. 82071390 to R J), the Tianjin Research Program of Application Foundation and Advanced Technology (no. 19YFZCSY00650 to R J), the Clinical Study of Tianjin Medical University (no. 2017kylc007 to R J), National Natural Science Foundation of China (no. 82071402 to W Q), and National Natural Science Foundation of Tianjin (no. 20JCYBJC01380 to W Q).

## CONFLICT OF INTEREST STATEMENT

The authors declare no conflicts of interest.

## Data Availability

The data that support the findings of this study are available from the corresponding author upon reasonable request.
